# Molecular Docking and Dynamic Simulation Revealed the Potential Inhibitory Activity of Opioid Compounds Targeting the Main Protease of SARS-CoV-2

**DOI:** 10.1155/2022/1672031

**Published:** 2022-12-21

**Authors:** Samaher S. A. Mahmoud, Eslam B. Elkaeed, Aisha A. Alsfouk, Elshimaa M. N. Abdelhafez

**Affiliations:** ^1^Clinical Pharmacy Program, Faculty of Pharmacy, Minia University, Minya 61519, Egypt; ^2^Department of Pharmaceutical Sciences, College of Pharmacy, AlMaarefa University, Riyadh 13713, Saudi Arabia; ^3^Department of Pharmaceutical Sciences, College of Pharmacy, Princess Nourah Bint Abdulrahman University, P.O. Box 84428, Riyadh 11671, Saudi Arabia; ^4^Medicinal Chemistry Department, Faculty of Pharmacy, Minia University, Minya, Egypt 61519

## Abstract

Opioids are a class of chemicals, naturally occurring in the opium poppy plant, and act on the brain to cause a range of impacts, notably analgesic and anti-inflammatory actions. Moreover, an overview was taken in consideration for SARS-CoV-2 incidence and complications, as well as the medicinal uses of opioids were discussed being a safe analgesic and anti-inflammatory drug in a specific dose. Also, our article focused on utilization of opioids in the medication of SARS-CoV-2. Therefore, the major objective of this study was to investigate the antiviral effect of opioids throughout an in silico study by molecular docking study to fifteen opioid compounds against SARS-CoV-2 main protease (PDB ID 6LU7, M^pro^). The docking results revealed that opioid complexes potentially inhibit the M^pro^ active site and exhibiting binding energy (-11.0 kcal/mol), which is comparably higher than the ligand. Furthermore, ADMET prediction indicated that all the tested compounds have good oral absorption and bioavailability and can transport via biological membranes. Finally, M^pro^-pholcodine complex was subjected to five MD (RMSD, RMSF, SASA, Rg, and hydrogen bonding) and two MM-PBSA, and conformational change studies, for 100 ns, confirmed the stability of pholcodine, as a representative example, inside the active site of M^pro^.

## 1. Introduction

Multiple instances of pneumonia with an unclear origin were reported in December 2019. A WHO-designated pathogen coronavirus illness was identified in 2019 (COVID-19) [1].

Laterally, it was renamed severe acute respiratory syndrome coronavirus two (SARS-CoV-2), which belonged to a family called *Coronaviridae* [2]. SARS-CoV-2 is a single-stranded RNA virus that is enclosed and has a positive sense (+ssRNA) [1].

### 1.1. Complications of COVID-19

Although there is no evidence of direct insult to the CNS, the virus was not detected in most CSF examinations [3]. No direct evidence of the virus in the brain has been discovered during postmortem examinations [4]. Individuals with COVID-19, in contrast, had a considerably higher rate of ischemic strokes than those with influenza [5]. There are not enough high-quality cohort studies or case series to get the whole picture.

As a complication of the neuromuscular system, death was reported in one of the 11 patients [6]. One of the fatal complications is thrombus formation due to SARS-propensity CoV-2 infecting endothelial cells via ACE-2 (angiotensin-converting enzyme 2) [7].

### 1.2. Opioids in the Treatment of SARS-CoV-2

Opioids have respiratory system effects like lowering the respiratory response CO_2_ [8], hypoxia [9], loading inspiratory flow-resistive [10], and exercise [11], with overdoses able to cause respiratory depression [12]. Opioid receptors are expressed on several immune system cells (monocytes, macrophages, lymphocytes, and neutrophils), decreasing lymphocyte proliferation and cytokine production [13, 14].

Morphine has been proven in both in vitro and in vivo animal studies to have various properties such as anti-inflammatory, antifibrotic, anticancer, cardioprotective, and renoprotective [15, 16]. Opioids can antagonize the impact of angiotensin-converting enzyme 2 (ACE2) [17], which results in the renin-angiotensin system being dysregulated (RAS). Additionally, opioids have been found to inhibit COVID-19 pathogenesis via cytokine production and inflammatory cell infiltration in the lungs during various viral infections by their immunomodulatory impact [18].

### 1.3. Opioid Family

**Scheme 1:**
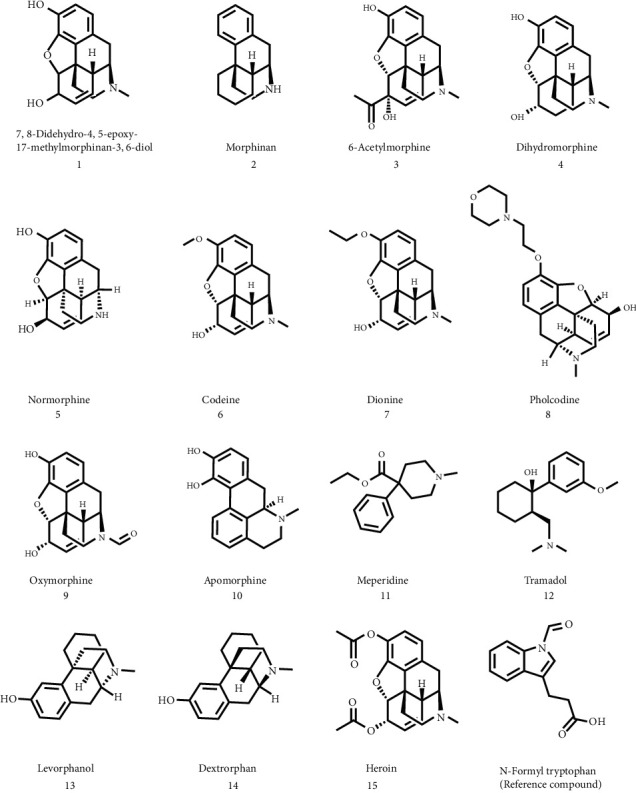
Structures of opioid compounds **1**-**15**.

Morphine (**2**), as a powerful analgesic and natural narcotic compound, is considered a powerful agonist of the u-opioid receptor with high abuse potential. Compound **2** is absorbed orally with a median time to the maximum blood concentration of 0.75 h [19]. According to recent studies, heroin (**15**) is a more potent analgesic than its more active metabolite morphine (**2**) and 6-acetylmorphine (**3**). Deacetylation of compound **15** to compounds **3** and **2** leads to questions about the exact cause responsible for the potency difference [20]. Compound **15** (diacetylmorphine) is a semisynthetic derivative of compound **2**. Compound **15** is lipid-soluble and absorbed rapidly after parenteral administration [21]. Compound **15** is biotransformed to compound **3** and is much slower than compound **2** by blood and various tissue, including the brain [22, 23]. Compared to compound **2**, compound **15** has a greater water solubility [20, 24], is the fastest onset of action [20], produces a greater degree of euphoria, and has fewer side effects. For example, compound **15** is approximately 2 to 16 times more potent than compound **2** in producing reinforcing effects in animals and subjective effects [25, 26]. We have discovered that compound **2**, but not compound **15**, binds to opiate receptors, suggesting that compound **15** serves primarily as a lipid-soluble prodrug for compound **2**'s central distribution. The discovery was that compound **2** levels in the brain are higher after compound **15** administration compared to a comparable dose of compound **2**. The 6-acetylmorphine possessed intrinsic activity and triggered several opiate-like effects [27, 28]. Studies suggested that compound **15** is rapidly hydrolyzed to its 6-acetyl derivative, and compound **3** has opiate receptor affinity, whereas compound **15** does not. As a result, compound **15** increased potency compared to compound **2** [20]. Codeine (**6**), also known as 3-methylmorphine, is a mild opioid with analgesic and antitussive properties [29]. Compound **6** is conversed to compound **2** by the cytochrome P450 enzyme, CYP2D6, which is responsible for its analgesic effect. Compound **6** also has some (low) affinity for the u-opioid receptor found in the central nervous system (CNS) and peripheral tissues such as the gastrointestinal tract [30]. When used as directed, low-dose compound **6** in fixed combinations with other drugs is effective and safe [31]. In animal test systems, pholcodine has antitussive activity comparable to compound **6** [32]. Normorphine (**5**) is an opiate analog that was first described in the 1950s as an N-demethylated derivative of morphine. Compound **6** is a less potent analgesic than compound **2**. An amount of 30 mg is indicated as a human dose that can produce less sedation, miosis, vomiting, and respiratory depression than an equal dose of compound **2** [33]. Meperidine (pethidine) (**11**) is a phenyl piperidine derivative that plays an opioid receptor agonist role. In the United States, meperidine is marketed under the brand name Demerol. Due to concerns regarding adverse effects, pharmacological interactions, and the neurotoxicity of normeperidine (its metabolite), several doctors are avoiding using this medication as an initial-line opioid analgesic [34]. Tramadol (**12**) is a racemic mixture of tramadol R (+) and tramadol S (-). Additionally, it regulates the monoaminergic system, unlike typical opioids, by reducing noradrenergic and serotoninergic reuptake [35]. As a result, tramadol is classified as an atypical opioid. Compound **12** is one of the most often recommended analgesics for moderate to severe pain owing to its special pharmacological features [36]. Compound **12** was established in Germany during the 1970s and received FDA approval in 1995 but was reclassified as a schedule IV drug in 2014 [34]. Other opioids are also used to treat pain. Other opioid analgesics and cough suppressants are also used to relieve pain. Ethylmorphine (**7**) is an opioid analgesic and cough suppressor. Norethylmorphine is demethylated to norethylmorphine (catalyzed by CYP3A4) and then O-deethylated to provide compound **2** (catalyzed by CYP2D6). Dihydromorphine (**4**) has been considered to be pharmacologically more effective because of its large selectivity for opioid receptors [19]. Levorphanol (**13**) is a one-of-a-type synthetic opioid due to its varied actions as an agonist for both the opioid and d- and k-opioid receptors. Compound **13** is also an antagonist of the N-methyl-D-aspartate receptor and a norepinephrine and serotonin reuptake inhibitor. The analgesic impact lasts between 6 and 15 hours [19]. 7,8-Didehydro-4,5-epoxy-17-methylmorphinan-3,6-diol (**1**) is derivative from epoxymorphinan which has sharp analgesic effect [37]. Compound (**1**) has general depressing activity consequently intended to be used in interfere with seizure threshold [38]. Based on the previously mentioned aspects, we encouraged to test the target 15 opioid compounds computationally as anti-SARS-CoV-2 candidates using molecular docking calculations as well as dynamic simulation for the most active candidates in addition to in silico ADMET prediction studies (Scheme 1).

## 2. Materials and Methods

### 2.1. Molecular Docking Study

The target opioid derivatives **1**-**15**(7,8-didehydro-4,5-epoxy-17-methylmorphinan-3,6-diol, morphine, 6-acetylmorphine, dihydromorphine, normorphine, codeine, ethylmorphine, pholcodine, oxymorphine, apomorphine, meperidine, tramadol, levorphanol-3-hydroxy-N-methyl-morphine, dextrophenol-3-hydroxy-N-methyl-morphine, and heroin) were docked against SARS-CoV-2 main protease (PDB: 6LU7, M^pro^) by using Molecular Operating Environment (MOE) version 2014.09. The M^pro^ was optimized and prepared for docking studies at first; then, the molecular docking has been run. Energy minimization was applied to all conformers, and all minimizations were carried out using MOE with the MMFF94X force field up to an RMSD gradient of 0.01 kcal/mol and RMS (root mean square) distance of 0.1. Partial charges were then automatically generated. Molecular Database (MDB) file was used to store the collected database utilized when making docking calculations.

#### 2.1.1. Optimization of M^pro^

The protein data bank provided the X-ray crystallographic structure of the binding site of M^pro^ (PDB: 6LU7). The chemicals were docked to the target enzyme's active site.

#### 2.1.2. Preparation of M^pro^

Delete the cocrystallized ligand. The system was then filled with conventional geometry hydrogen atoms. Automatic correction was used to check for flaws in the atoms' connection and type. The receptor's choice and atom's potential were fixed.

#### 2.1.3. Docking of the 15 Molecules to M^pro^ Active Site

The MOE-Dock software was used to dock the target molecules. In general, the following methods were used.

The Dock tool was launched after loading the enzyme active site file. The program's requirements were changed to include the following:

(i) Dummy atoms as the docking site
(ii) Triangle Matcher will be utilized as the placement approach
(iii) London dG was chosen as the scoring mechanism, and its default parameters were set
  - Dock calculations were automatically performed after loading the MDB file of the ligand that needed to be docked
  - After studying the acquired poses, the poses that best represented the interactions between the ligand and the enzyme were chosen and saved for energy calculations

### 2.2. MD Simulations

CHARMM-GUI web interface and CHARMM36 force field were used to prepare the M^pro^-pholcodine complex. The NAMD 2.13 package was used for all of the simulations. The periodic boundary conditions were set with a dimension of certain dimensions in *x*, *y*, and *z*, respectively, and the TIP3P explicit solvation model was utilized. The CHARMM general force field was used to produce the parameters for the best docking findings. After that, (Cl-/Na+) ions were used to neutralise the system. Production, equilibration, and minimization were all part of the MD protocols. All MD simulations used a 2 fs time step of integration, with the canonical (NVT) ensemble used for equilibration and the isothermal-isobaric (NPT) ensemble used for production. The pressure was maintained at 1 atm using a Nose-Hoover Langevin piston barostat with a Langevin piston decay of 0.05 ps and a period of 0.1 ps throughout the 100 ns of MD generation. The Langevin thermostat was used to set the temperature at 298.15 K. The Lennard-Jones interactions were smoothly trimmed at 8.0, and a distance cut-off of 12.0 was applied to short-range nonbonded interactions with a pair list distance of 16. The particle-mesh Ewald (PME) approach was utilized to handle long-range electrostatic interactions, with a grid spacing of 1.0 being applied to every simulation cell. The SHAKE method was used to restrict all hydrogen atom covalent bonds. We used the same protocol for all MD simulations in order to maintain consistency.

### 2.3. Physicochemical Properties and Lipophilicity

A wide range of cheminformatics tools supporting the manipulation and processing of molecules are available from SwissADME and Molinspiration, including the conversion of SMILES and SD files, normalisation of molecules, production of tautomers, molecule fragmentation, calculation of various molecular properties required in QSAR and drug design, high-quality molecule depiction, and molecular database tools supporting substructure and similarity searches. Additionally, these tools allow data visualisation, bioactivity prediction, and fragment-based virtual screening. Because Molinspiration tools are designed in Java, they can essentially run on any platform. In order to eliminate structures with unsuitable properties for drugs and choose promising drug candidates, calculated molecular descriptors may be utilized for property-based virtual screening of vast collections of molecules. The following molecular characteristics were determined using Molinspiration and SwissADME.

### 2.4. Drug-Likeness Calculation on the Basis of Lipinski's Rule of Five

ChemBioDraw Ultra program (11.0 version) was used to get the chemical structures and SMILES notations of the opioid derivatives **1** through **15**. When using Molsoft and SwissADME to calculate breaches of Lipinski's rule of five and the bioavailability score to assess the drug similarity, SMILES notations of the opioid derivatives **1**–**15** are fed in. The Lipinski rule of five, which states that any compound considered to be a drug should have a partition coefficient less than 5, a polar surface area within 140 2, an H-bond acceptor less than 10, an H-bond donor less than 5, and a molecular weight within 500 dalton, is the foundation for the calculation of these properties.

### 2.5. ADME Data of Tested Compounds

Using the SwissADME software, the ADMET descriptors (absorption, distribution, metabolism, excretion, and toxicity) of the opioid derivatives **1**–**15** were identified. The studied compounds were first produced and minimized in accordance with the synthesis of small molecule methodology, after which the CHARMM force field was applied. Models for human intestinal absorption, aqueous solubility, blood-brain barrier penetration, plasma protein binding, cytochrome P450 (CYP1A2, CYP2C19, CYP2C9, and CYP3A4) inhibition, and skin permeability were among the ADMET descriptors that were included in the application.

## 3. Results and Discussion

### 3.1. Docking Studies

A reasonable approach to tackle the supposed anti-COVID-19 activity hypothesis is to investigate the possible binding energies and modes for the opioid derivatives **1**-**15** and reference to assume the binding interactions between them and the SARS-CoV-2 main protease, M^pro^, binding site that was downloaded from the website of protein data bank under the code (PDB: 6LU7). The docking studies were performed using the software Molecular Operating Environment (MOE) version 2014.09.

**Figure 1:**
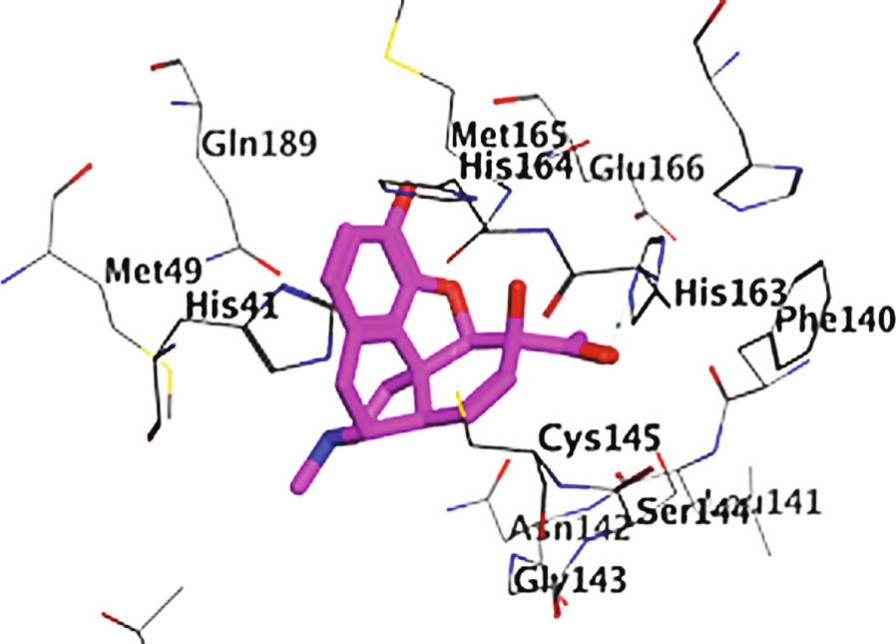
2D and 3D binding modes of 3 interacted with the M^pro^ active site (PDB ID 6LU7).

**Figure 2:**
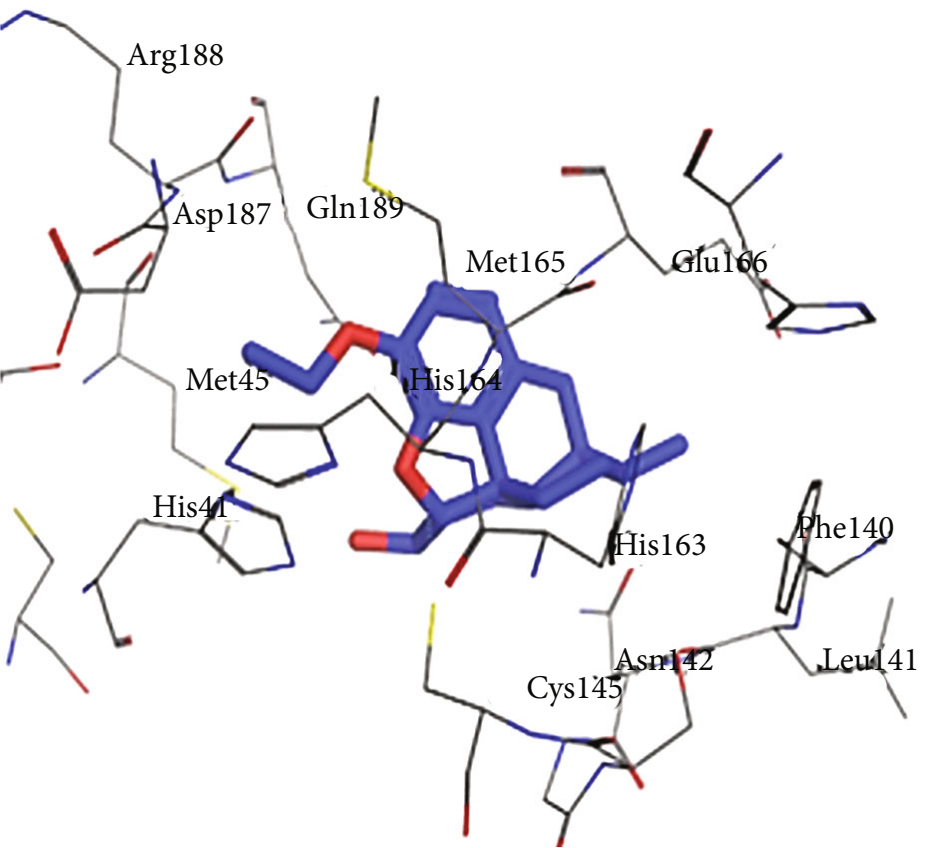
2D and 3D binding modes of 7 interacted with the M^pro^ active site (PDB ID 6LU7).

**Figure 3:**
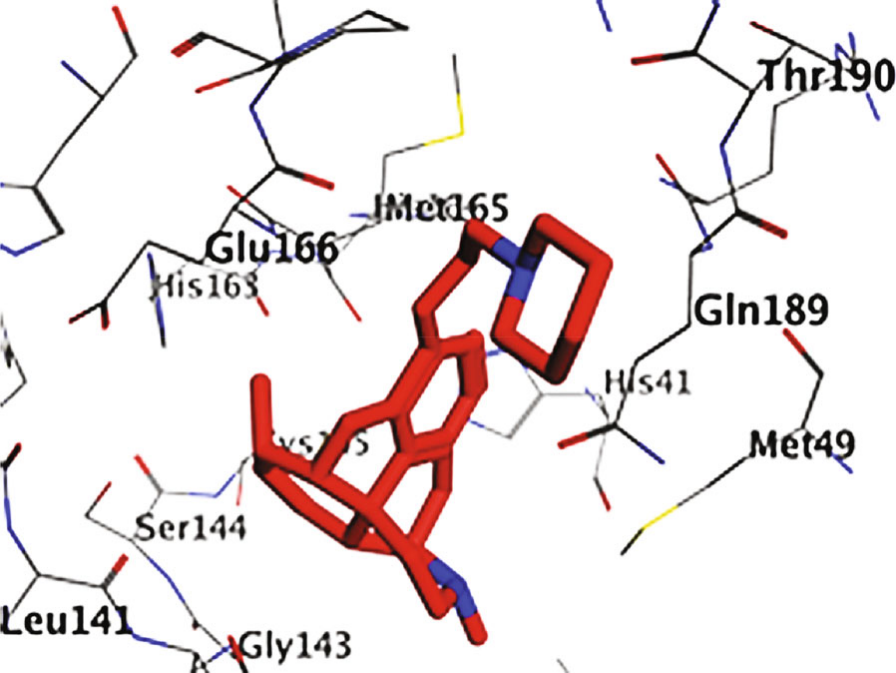
2D and 3D binding modes of **8** interacted with the M^pro^ active site (PDB ID 6LU7).

All opioid derivatives, **1**-**15**, were successfully docked into the M^pro^ binding pocket. The most favorable poses as well as the binding free energies (Δ*G*) of that poses of the opioid derivatives (**1**-**15**) are shown in Figures 1–3 and listed in Table 1, respectively. Most of the opioid derivatives **1**-**15** exerted high binding affinity to the M^pro^ as their Δ*G* values range from -0.5 to -5.3 kcal/mol, compared to the reference (Δ*G* = −1.0 to -1.2 kcal/mol).

**Figure 4:**
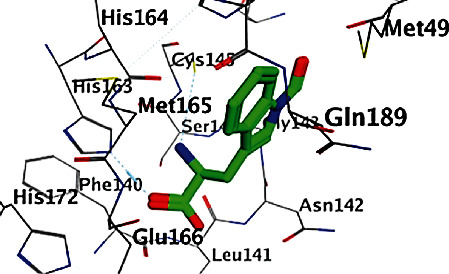
2D and 3D binding modes of ligand interacted with the M^pro^ active site (PDB ID 6LU7).

The docking result of reference compound **1** is completely consistent with that obtained for opioid derivatives (Figure 4). The 2D diagram showed a crucial binding with hydrogen bonding interaction with MET165 and hydrophobic interaction with GLU166 amino acid residues.

Docking results with the M^pro^ of opioid derivatives **1**-**15** revealed that most of the opioid compounds showed good binding with the M^pro^ making several vital interactions comparing the reference and the most active compounds **3**, **7**, and **8** (Figures 1–3). Compounds **2**, **4**, **7**, **8**, **10**, **12**, **13**, and **14** exhibited hydrophobic interaction with the conserved amino acid GLU166, typically as the reference. On the other hand, none of the tested compounds interacted with MET165 amino acid residue.

Additionally, compounds **1**, **3**, **10**, and **15** showed hydrogen bonding as (an H-acceptor) interaction with the HIS163 amino acid residue (Figure 1 for compound **3**). Moreover, extra binding more than the reference, such as compounds **5** and **10**, showed interactions within the binding site of the M^pro^, revealing hydrogen bonding and hydrophobic pi-H interaction with SER144 and ASN142 amino acid residues, respectively, for compounds **5**, **6**, and **9**; however, compound **6** lacks interaction with SER144 amino acid. The opioid derivatives **10** and **11** possess off-binding interaction and hydrogen bonding interaction with GLN189 and GLY143 amino acid residues, respectively.

### 3.2. Molecular Dynamics (MD) Simulations

**Figure 5:**
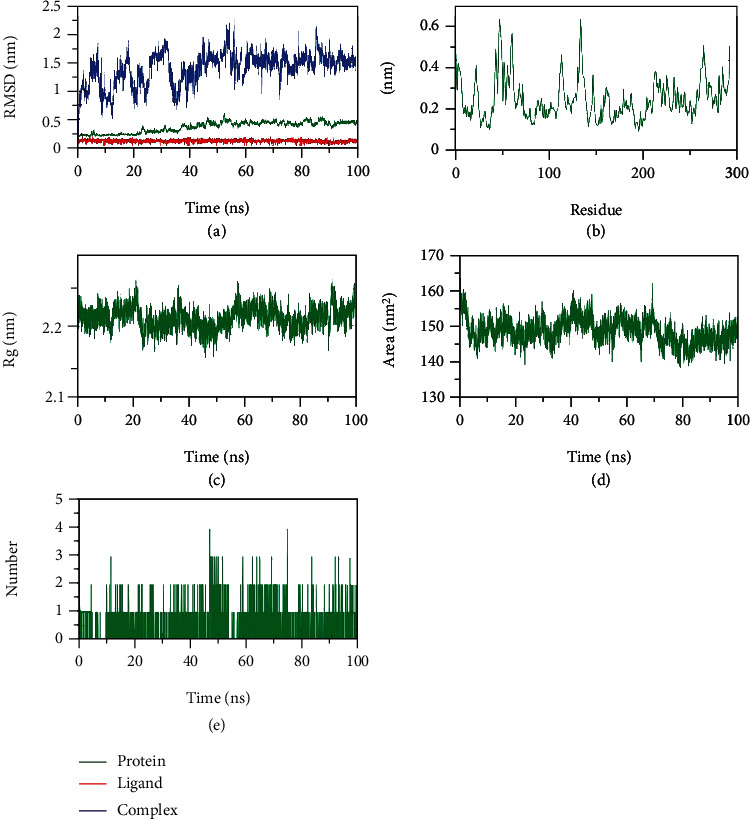
MD outputs of the M^pro^-pholcodine complex: (a) RMSD, (b) RMSF, (c) R_g_, (d) SASA, and (e) configuration of H-bonds.

Pholcodine, compound **8**, exhibited an excellent binding mode against the M^pro^, and accordingly, it has been selected for further studies. The changes in the conformations of the M^pro^-pholcodine complex, M^pro^, and pholcodine, in addition to their energies in both apo and combined states, were studied on an atomic level through the calculation of the RMSD values (Figure 5(a)). The M^pro^-pholcodine complex expressed a minor level of fluctuation till ~60 ns and stabilized later till the end of simulations (100 ns). The amino acids' flexibility of M^pro^ was examined in terms of RMSF to figure out the protein's region that fluctuated through the process of simulation. As Figure 5(b) demonstrates, the binding of pholcodine does not make M^pro^ flexible. The solidity and stability of the M^pro^-pholcodine complex were investigated by the computation of the radius of gyration (Rg) that is inversely proportional to both solidity and stability. Figure 5(c) indicates that the Rg of the M^pro^-pholcodine complex at 100 ns was almost similar to that at 1 ns. The solvent accessible surface area (SASA) was computed over 100 ns to estimate the interactions between the M^pro^-pholcodine complex and the solvents in the media. As illustrated in Figure 5(d), the M^pro^-pholcodine complex featured a decrease in the surface area as the SASA values were computed to be lower at the end of the study than at the start. The hydrogen bonding level of the M^pro^-pholcodine complex was computed, and the highest number of hydrogen-bond conformations of the M^pro^ formed up to three hydrogen bonds with pholcodine (Figure 5(e)).

### 3.3. MM-PBSA

**Figure 6:**
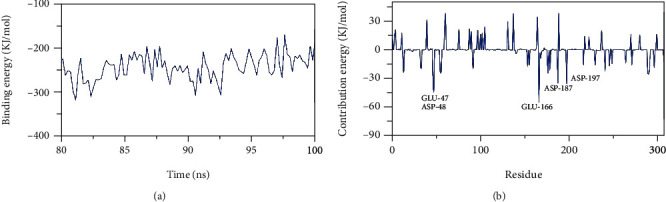
MM-PBSA analysis of M^pro^-pholcodine complex: (a) free energy of binding; (b) analyzed energy of binding.

By calculating the average free binding energy from MD trajectories with a time interval of 100 ps, we were able to determine that the pholcodine has an extremely low binding free energy of -243 KJ/mol with the M^pro^ and that the binding energy was stable over the whole period of our analysis, showing accurate binding (Figure 6(a)).

Next, we analyzed the total binding free energy of the M^pro^-pholcodine complex to elucidate the different parts of the binding energy and to reveal which amino acid residues played a major role in binding the ligand to the M^pro^ to identify which amino acids had the most favorable impact in binding.  Figure 6(b) illustrates that five amino acid residues of the M^pro^ (GLU-47, ASP-48, GLU-166, ASP-187, and ASP-197) contributed more than -30 kJ/mol binding energy and are therefore considered hotspot residues in binding.

### 3.4. Conformational Changes

**Figure 7:**
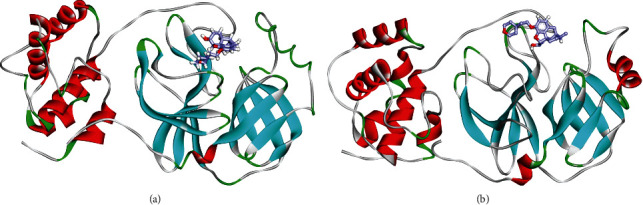
Ribbon diagram showing the conformational changes of the M^pro^-pholcodine complex during the 1st (a) and 100th ns (b) of the MD production run.

Figure 7 illustrates the conformational changes that occurred because of the binding of the M^pro^-pholcodine complex during the 1st and 100th ns of the MD production run, indicating the incidence of some conformational changes. Additionally, the binding stability and the integrity of the complex were confirmed as pholcodine kept binding firmly to the M^pro^ throughout the study.

### 3.5. In Silico Prediction of Drug-Likeness Profiles

The design and applied new drugs are complicated because of the unacceptable ADMET parameters (distribution, excretion, absorption, metabolism, and toxicity) and the high costs for new drug development. Accordingly, it is critical to estimate the ADMET properties of a new drug [39]. Recently, the in silico ADMET analysis was applied vastly, decreasing the degradation in late production stages [40, 41]. Many parameters, such as the aqueous solubility, polar surface PSA, partition coefficients, cell permeability, and intestinal absorption, have been investigated in several virtual screening studies. Lipinski's rule links the good level of oral bioavailability of a certain drug with different parameters. The molecular weight (M Wt.), Log *P*, hydrogen bond (HB) acceptor atoms, and HB donor atoms should be >500, >5, >10, and >5, respectively [42]. The rotatable bond's number indicates molecular flexibility that is essential in oral bioavailability. The percentage absorption (% ABS) was found to be inversely proportional to the polar surface area (PSA) measured by the equation %ABS = 109 − 0.345 tPSA [39].

Herein, we employed the software of Molinspiration [43], Molsoft [44], and SwissADME [39] to predict the ADMET characteristics of the examined opioid candidates.  Table 2 shows that all compounds except compound **10** obey Lipinski's rule with Log *P* range values 1.09-3.36 (<5), MW range 247.33-398.50 (<500), HBD from 0 to 3 (≤5), and HBA from 1 to 6 (<10) (Table 3). The examined compounds would have a high-level oral absorption. Also, the values of the topological PSA were in the range of 12.47-70.00 A^2^ (<140 A^2^), and the oral absorption percentage ranges were 84.85 to 104.84%, indicating high levels of absorption, permeability, and biological membrane transport. Also, the drug-likeness profiles of the examined candidates were verified by the Molsoft software (Table 2). The compounds exhibited values of solution ability specifications ranging from 1.08 to 8.38 mg/L (more than 0.0001 mg/L). A positive model scores between 0.29 and 1.23 were predicted for all the tested candidates except compound **3**.

Additionally, some other pharmacokinetic parameters were evaluated using the SwissADME software as follows: GIT absorption level, P-gp substrate, and the inhibitory potential against several cytochrome P-450 targets. The results are listed in Table 4. The investigated opioid candidates showed medium to low ability in the skin permeability model with Log *Kp* ranging from -5.51 to 8.16. Also, all compounds exerted high level of human intestinal absorption. Most of the examined compounds **2**-**7**, **10**, **11**, **14**, and **15** were highly bound to human glycoproteins.

## 4. Conclusion

In the global pandemic of the SARS-CoV-2, a huge need for a new treatment modality has been emerged. Our molecular docking studies revealed that the tested opioid (**1**-**15**) had a better binding affinity with the SARS-CoV-2 M^pro^ active site (PDB ID 6LU7) than their corresponding reference and might be better alternatives to prevent SARS-CoV-2 and warrant further in vitro*/*in vivo application of the opioid candidates against SARS-CoV-2. Moreover, the results of testing pharmacokinetic and physicochemical parameters showed that compounds **2**-**15** stratify to Lipinski's rule and indicate good pharmacokinetic parameters. Finally, MD, MM-PBSA, and conformational studies were conducted and indicated the stability of pholcodine, as a representative example, inside M^pro^ for 100 ns. Therefore, these opioid compounds are predicted to be promising and potent anti-SARS drug discovery.

## Figures and Tables

**Table 1:** Energy scores for the complexes formed by the tested compounds **2**-**15** and reference [1] in the active site of the SARS-CoV-2 M^pro^ enzyme (PDB: 6LU7).

Code	Compounds	*S* score	Residue	Type of interaction	Δ*G* (kcal/mol)	Length (Å)
	Ligand	-6.703	MET 165	H-donor	-1.0	3.70
			GLU 166	Pi-H	-1.2	4.68
1	7,8-Didehydro-4,5-epoxy-17-methylmorphinan-3,6-diol	-4.920	HIS 163	H-acceptor	-4.6	3.07
2	Morphine	-4.074	GLU 166	H-donor	-1.0	2.99
3	6-Acetylmorphine	-5.672	HIS 163	H-acceptor	-1.2	3.54
4	Dihydromorphine	-5.005	GLU 166	Pi-H	-1.9	4.10
5	Normorphine	-4.817	SER 144	H-donor	-0.5	2.96
			ASN 142	Pi-H	-0.6	4.36
6	Codeine	-5.563	ASN 142	Pi-H	-0.6	4.69
7	Ethylmorphine	-5.824	GLU 166	Pi-H	-1.7	4.26
8	Pholcodine	-5.738	GLU 166	Pi-H	-0.8	4.51
9	Oxymorphine	-5.010	SER 144	H-donor	-0.5	2.97
			ASN 142	Pi-H	-0.6	4.37
10	Apomorphine	-4.965	HIS 41	H-Pi	-2.4	3.54
			GLU 166	Pi-H	-0.6	4.81
			GLN 189	Pi-H	-1.0	4.47
11	Meperidine	-5.246	GLY 143	H-acceptor	-1.6	3.18
12	Tramadol	-5.229	GLU 166	Pi-H	-0.7	4.59
13	Levorphanol-3-hydroxy-N-methyl-morphine	-5.00	GLU 166	Pi-H	-1.7	4.35
14	Dextrophenol-3-hydroxy-N-methyl-morphine	-5.216	GLU 166	Pi-H	-1.5	4.44
15	Heroin	-5.465	HIS 163	H-acceptor	-11.0	2.89
			HIS 163	ionic	-5.3	2.89

**Table 2:** Lipinski's drug-likeness of the opioid candidates.

Comp.	Solubility (mg/L)	Drug-likeness model score	Lipinski's violations	Bioavailability score
1	1.76	0.76	0	0.55
2	2.45	0.73	0	0.55
3	4.01	-0.15	0	0.55
4	8.19	1.00	0	0.55
5	8.38	0.77	0	0.55
6	1.08	0.14	0	0.55
7	5.15	0.86	0	0.55
8	2.16	1.03	0	0.55
9	1.97	1.49	0	0.55
10	7.13	0.33	0	0.55
11	1.16	0.38	0	0.55
12	2.57	1.23	0	0.55
13	3.37	0.95	0	0.55
14	3.67	0.96	0	0.55
15	7.74	0.29	0	0.55

**Table 3:** Physicochemical properties and lipophilicity of the opioid compounds (**1**-**15**).

Physicochemical properties										
Compound	Lipophilicity consensus Log *P*	MW^i^	HA^ii^	AHA^iii^	Rot. bond	HB acc.	HB don.	MR^iv^	TPSA^v^ (A^2^)	^vi^ABS^∗∗∗∗^
1	2.20	369.41	27	6	4	6	0	101.48	65.07	86.55
2	1.42	285.34	21	6	0	4	2	82.27	52.93	90.74
3	3.16	277.34	17	6	0	1	1	75.09	12.03	104.84
4	1.28	327.37	24	6	1	5	2	92.12	70.00	84.85
5	1.76	287.35	21	6	0	4	2	82.74	52.93	90.74
6	1.09	271.31	20	6	0	4	3	77.37	61.72	87.71
7	1.75	299.36	22	6	1	4	1	86.74	41.93	94.53
8	2.12	313.39	23	6	2	4	1	91.55	41.93	94.53
9	1.64	398.50	29	6	4	6	1	83.02	54.40	90.23
10	7.13	299.32	22	6	1	4	2	116.56	70.00	84.85
11	2.47	267.32	20	12	0	3	2	82.86	43.70	93.92
12	2.53	247.33	18	6	4	3	0	75.73	29.54	98.81
13	2.60	263.38	19	6	4	3	1	78.18	32.70	97.72
14	2.97	257.37	19	6	0	2	1	82.01	23.47	100.90
15	3.36	271.40	20	6	1	2	0	86.48	12.47	104.69

MW^i^: molecular weight (g/mol); HA^ii^: heavy atoms; AHA^iii^: aromatic heavy atoms; MR^1v^: molar refractivity; TPSA^v^: topological polar surface area; %ABS^vi^: absorption percentage.

**Table 4:** ADME data of the opioid candidates.

Comp.	Pharmacokinetics								
	GI^a^	BBB^b^	P-gp^c^	CYP1A2 inhibitor	CYP2C19 inhibitor	CYP2C9 inhibitor	CYP2D6 inhibitor	CYP3A4 inhibitor	Log *Kp*(skin permeation)
1	H	Yes	No	No	No	No	Yes	Yes	-7.43
2	H	Yes	Yes	No	No	No	Yes	No	-7.50
3	H	Yes	Yes	No	No	No	Yes	No	-5.56
4	H	No	Yes	No	No	No	Yes	No	-7.94
5	H	Yes	Yes	No	No	No	Yes	No	-6.75
6	H	No	Yes	No	No	No	Yes	No	-8.08
7	H	Yes	Yes	No	No	No	Yes	No	-7.32
8	H	Yes	No	No	No	No	Yes	No	-7.14
9	H	No	No	No	No	No	Yes	No	-8.18
10	H	No	Yes	No	No	No	Yes	No	-8.29
11	H	Yes	Yes	Yes	No	No	Yes	No	-6.30
12	H	Yes	No	No	No	No	Yes	No	-5.88
13	H	Yes	No	No	No	No	Yes	No	-6.10
14	H	Yes	Yes	No	No	No	Yes	No	-5.66
15	H	Yes	Yes	No	No	No	Yes	No	-5.51

GI^a^: absorption from the gastrointestinal tract; BBB^b^: penetration of the blood-brain barrier; P-gp^c^: P-glycoprotein substrate.

## Data Availability

The computed and calculated data used to support the findings of this study are included in the article.
